# Proton Transport Chains in Glucose Metabolism: Mind the Proton

**DOI:** 10.3389/fnins.2018.00404

**Published:** 2018-06-15

**Authors:** Dirk Roosterman, Wolfgang Meyerhof, Graeme S. Cottrell

**Affiliations:** ^1^Ruhr-Universität Bochum, Bochum, Germany; ^2^Center for Integrative Physiology and Molecular Medicine, Saarland University, Homburg, Germany; ^3^School of Pharmacy, University of Reading, Reading, United Kingdom

**Keywords:** glycolysis/gluconeogenesis, lactate dehydrogenases, GAPDH (glyceraldehyde-3-phosphate dehydrogenase), proton transport chain, pyruvate dehydrogenase complex, monocarboxylic acid transporters, phosphoglycerate kinase

## Abstract

The Embden–Meyerhof–Parnas (EMP) pathway comprises eleven cytosolic enzymes interacting to metabolize glucose to lactic acid [CH_3_CH(OH)COO**H**]. Glycolysis is largely considered as the conversion of glucose to pyruvate (CH_3_COCOO^-^). We consider glycolysis to be a cellular process and as such, transporters mediating glucose uptake and lactic acid release and enable the flow of metabolites through the cell, must be considered as part of the EMP pathway. In this review, we consider the flow of metabolites to be coupled to a flow of energy that is irreversible and sufficient to form ordered structures. This latter principle is highlighted by discussing that lactate dehydrogenase (LDH) complexes irreversibly reduce pyruvate/H^+^ to lactate [CH_3_CH(OH)COO^-^], or irreversibly catalyze the opposite reaction, oxidation of lactate to pyruvate/H^+^. However, both LDH complexes are considered to be driven by postulated proton transport chains. Metabolism of glucose to two lactic acids is introduced as a unidirectional, continuously flowing pathway. In an organism, cell membrane-located proton-linked monocarboxylate transporters catalyze the final step of glycolysis, the release of lactic acid. Consequently, both pyruvate and lactate are discussed as intermediate products of glycolysis and substrates of regulated crosscuts of the glycolytic flow.

## Introduction

A dysregulated glucose/lactate ratio in blood or spinal fluid is an acknowledged finding in chronic diseases coupled with inflammatory processes, such as diabetes, hypertension, schizophrenia, major depression or multiple sclerosis (Juraschek et al., 2013, 2015; Rowland et al., 2016; Pucino et al., 2017). The glycolytic pathway is one of the best-investigated pathways in biology. However, have a century of investigations failed to provide any clues about glucose metabolites regulating homeostasis (Steiner et al., 2014)?

Glycolysis is thought of as a tool that provides ATP and not as ancient pathway regulating life; glucose, lactic acid and carbon dioxide are all understood as substrates or end products of either “anaerobic” or “aerobic” glycolysis, respectively. They are not considered as primary messengers communicating the energy state of a cell to the organism.

Mathematical models were developed describing the interaction of the eleven cytosolic enzymes of the EMP pathway in a steady state situation (Kacser and Burns, 1973; Rapoport et al., 1974, 1976; Westerhoff et al., 1987). The current ‘steady state’ model developed to describe glucose metabolism is based on random movement and random location of molecules in the cytosol, or put simply ‘chaos.’ The premise of the steady state concept is the random distribution of the molecules in the cytosol, which mathematically and in chemical formulae is defined as [mol/L]. However, this cannot be true in a cell, as the concentration of an intermediate product is highest at the catalyzing enzyme or the place where the substrate enters the cell. Moreover, in an ordered system, chaos cannot recognize or determine the destiny of molecules, neither can it sense or determine the destiny of molecules. We believe that a model based on chaos is inadequate to regulate and detect cellular energy metabolism.

This review discusses that once proteins are embedded in ordered structures, enzymatic reactions turn from reversible to irreversible processes. The reaction product of one enzyme becomes the substrate for another enzyme in the immediate proximity. As such, the substrate concentration has to be considered as infinite. Infinite, because the substrate is bound within the active site of the enzyme. Enzyme-bound substrate is free of water, thus the concentration of the substrate [mol] in water [L] must be calculated by dividing by the volume of water, zero, therefore infinite. The concentration remains infinite, when the proximity of the enzymes excludes water. The substrate is catalyzed to and released as intermediate substrate within a complex, where neither the movement nor location are random. Thus, we have developed mathematical model based on substrate provision (mol/s). The irreversible flow of metabolites (mol/s) is continuously sensed by these glycolytic protein complexes. Changes in the flow are detected, amplified and transmitted to signaling cascades. Otto Meyerhof demonstrated that glycogen was the precursor of lactic acid in isolated muscles (Meyerhof, 1927). The glycolytic pathway was elucidated by the early 1940s showing that eleven enzymes interact to catalyze the conversion of glucose to lactic acid (lactate and H^+^) (Meyerhof, 1942). However, today’s consensus is that the ancient enzyme, LDH, is not considered an enzyme of the EMP pathway regardless of its functional expression in bacteria, archaea or human cells (Oren and Gurevich, 1995; Bräsen et al., 2014). Consequently, instead of lactate, pyruvate occupies the place at the pivotal node in the regulation of carbon metabolism, as the end product of glycolysis and as the major substrate for the TCA cycle in mitochondria (Bricker et al., 2012; Herzig et al., 2012). In the LDH-truncated EMP pathway enzyme 10, pyruvate kinase, is believed to catalyze the final and rate-limiting step of glycolysis (Gupta and Bamezai, 2010). This popular and frequently propagated interpretation of the EMP pathway has become common knowledge and a topic already taught at school.

However, this scenario contradicts the principle of mass conservation and the original finding of Otto Meyerhof, illustrating that glucose (C_6_H_12_O_6_) is metabolized to two molecules of lactic acid (2x C_3_H_6_O_3_) (Meyerhof, 1927; Volkenstein, 2009). Additionally, ions do not diffuse through lipid bilayers. An enzymatically catalyzed membrane-transfer of monocarboxylates must be coupled with the co-transport of a cation or an anion exchange to establish the obligate charge-neutral transfer through lipid bilayers.

Contrary to the popular dogma that pyruvate is the end product of glycolysis and a unique substrate of the TCA cycle, it has been put forward that lactate is the major product of cerebral (and other tissues) glycolysis, under aerobic and anaerobic conditions, in neurons and astrocytes in resting state or during activation (Schurr, 2014). Consequently, it follows that the major glycolysis end product, lactate, serves as the major substrate for the mitochondrial TCA cycle (Schurr, 2014). Considering LDH as integral part of the glycolytic pathway and lactate (C_3_H_5_O_3_^-^) as the end product still does not obey to the principle of mass conservation (mind the proton, H^+^) presented in the original work of Meyerhof (1927).

Whereas the EMP pathway comprises only cytosolic enzymes, we consider glycolysis as a cellular process. Therefore, the transfer of glucose across the plasma membrane by GLUTs is the first enzyme-catalyzed step of the glycolytic process (Westerhoff et al., 2009). This step ensures a crucial function, namely the regulated provision of substrate. In an organism, blood glucose levels and cellular glucose uptake are tightly regulated processes. Increases in glucose levels can provoke the release of pro-inflammatory cytokines. Thus, clues exist considering glycolysis as signaling pathway (Collier et al., 2008; Gonzalez et al., 2012; Wang et al., 2012). We consider glucose as a “ligand” of an ancient signaling pathway called glycolysis. Whereas GLUTs perform the first step in glycolysis, proton-linked MCTs mediate the final step in line with the principle of mass conservation, i.e., the release of lactic acid into the environment (Halestrap, 2013). Moreover, we consider proton-linked MCTs, catalyzing the uptake of lactate and H^+^ and the release of lactic acid into the cytosol, as the first enzymes of another ancient signaling pathway called gluconeogenesis and lactate and H^+^ as “ligands” of this pathway. Thus, glycolysis starts with the regulated uptake of glucose from the environment into the cytosol and finishes with the release of lactic acid into the environment. Gluconeogenesis starts with the defined uptake of lactic acid. The differentiation between glycolysis and gluconeogenesis is only possible when both pathways are separated from each other by unidirectional acting enzyme complexes.

Our concept resets the understanding(s) of glycolysis back to the early 1940s. Pyruvate and lactate are considered as intermediate products rather than end products (Hui et al., 2017). The TCA cycle turns from a final destination for end product(s) to an alternative destination for the intermediate products of the metabolic pathway. The final product of glycolysis, lactic acid, is always released, under aerobic and anaerobic conditions, in neurons and astrocytes in resting state or during activation (Schurr, 2014). Consequently, lactic acid is not a product of (anaerobic) fermentation. Obligate aerobic multicellular organisms, such as humans, do not ferment.

Glucose metabolism provides energy/exergy (Grassmann, 1984). Here we argue that the reversible flow of metabolites is directionally driven by an irreversible flow of energy. The presented model of an energy flow-driven EMP, challenges the established steady state model (Kacser and Burns, 1973; Rapoport et al., 1974, 1976). Within this flow of energy, H^+^ is introduced as a reactive entity regulating directional metabolite flow or providing the energy turning reversible reaction into irreversible (Grassmann, 1984). The difference between enzymatically formed H^+^ and energetically stabilized hydronium [H^+^(H_2_O)_6_] is reviewed, leading to the postulation that the intermediate product of proton-linked MCT1-catalyzed membrane transfer actually is lactic acid and not lactate and H^+^. In thermodynamics, a flow of energy is considered to be sufficient to form ordered structures (Morowitz, 1992; Jorgensen, 1999; Orgel, 2000). In line with the tentative fourth law of thermodynamics (Morowitz, 1992), the ordered structures of the cytosolic GAPDH/LDH complex and the mLDH/proton-linked MCT1/PDH complex are discussed in terms of energy flow and as proton transport chains.

Integration of the mitochondrial-located TCA cycle as end product-regulated crosscut of intermediate products of the EMP pathway enticed us to split “The TCA cycle” into separate pyruvate-driven and lactate-driven TCA cycles. The discussed regulatory mechanisms of the TCA cycles suggest that anabolic (reducing) processes are coupled with the pyruvate-driven TCA cycle whereas the lactate-driven TCA cycle is coupled with the ETC.

## The Hydrogen Ion

**FIGURE 1:**
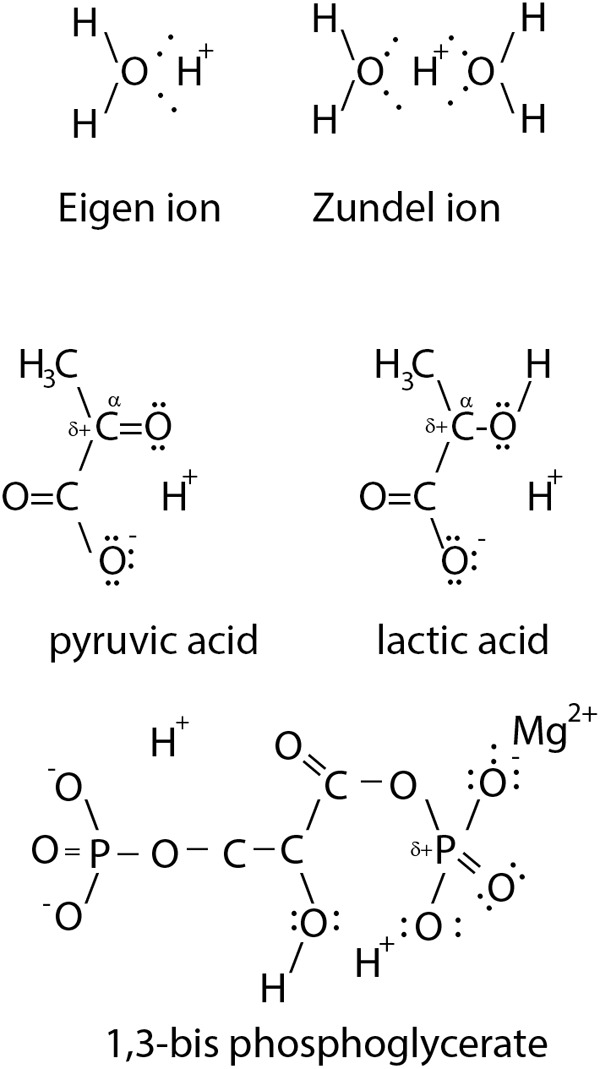
Illustration of a *di*solvated H^+^. In solution H^+^ is solvated (Eigen ion) usually *di*solvated by 2 H_2_O (Zundel ion). The α-C bound O-atom of monocarbonic acids, such as lactic acid and pyruvic acid, intra-molecularly *di*solvate the H^+^. The monocarbonic acids share the common characteristic of a partially positively charged (δ+) α-C atom to allow nucleophile substitution. Similarly, 1,3-bisphosphoglycerate uses O atoms to *di*solvate two protons.

A hydrogen ion (proton, H^+^) is highly reactive and just a stoichiometric result of reaction equations. H^+^ is always solvated, usually *di*solvated, which means that H^+^ is bounded to at least one O atom, or almost always two O atoms (Eigen, 1964; Zundel, 1976; Reed, 2013). The nature of H^+^ in water is thereby H^+^(H_2_O)_2_, which is further solvated by 4 H_2_O molecules to form a hydronium [H^+^(H_2_O)_6_] (Reed, 2013). The concentration of hydronium in the cytosol is approximately 10^-7^ M, indicating that hydronium is unlikely to act as a substrate for enzyme-catalyzed reactions due to both its low concentration and energetic stability. For simplicity H^+^ is normally written down as H^+^ in enzymatic reactions, but it must be *di*solvated, meaning that the nature of this new H^+^ is not defined. It is known, however, that lactic acid and pyruvic acid intra-molecularly *di*solvate H^+^ using their O-group located at the αC-atom (**Figure 1**). This intra-molecularly *di*solvated H^+^ is stabilized. This phenomenon is also illustrated for 1,3-bisphosphoglycerate (**Figure 1**). The pKa of H_2_PO_4_^-^/HPO_4_^2-^ is 7.2. Thus, at neutral pH, phosphophosphate groups mainly carry just one negative charge. Nonetheless, in chemical formulae 1,3-bisphosphoglycerate is commonly illustrated as carrying four negative charges. We suggest that 1,3-bisphosphoglycerate *di*solvates two H^+^ and 1,3-bisphosphoglycerate is better described as having two instead of four negative charges. PGK catalyzes a nucleophilic substitution. Therefore, the transferred 1-phosphate group has to be charge neutral, albeit with a partially positively charged P atom due to the pull of the neutralized O atoms on the P electrons. Mg^2+^ is present in the active site of PGK and likely plays a role in neutralizing the remaining negative charge of the 1-phosphate group of 1,3-bisphosphoglycerate to allow the nucleophilic substitution (Bernstein and Hol, 1998). Thus, PGK catalyzes 1,3-bisphosphoglycerate to 3-phosphoglycerate and H^+^. The product of the PGK catalyzed reaction is 3-phosphoglyceric acid.

Based on the nature of H^+^, it is reasonable to argue that the intermediate product of mLDH-catalyzed oxidation of lactate is pyruvic acid and not pyruvate and H^+^. Pyruvic acid *di*solvates the newly formed proton as a reactive H^+^. Similarly, the intermediate product of MCT-catalyzed membrane transfer of lactate and H^+^ is considered to be lactic acid and not lactate and H^+^. Released in water, monocarboxylic acids rapidly dissociate to energetically stabilized monocarboxylates and hydronium, formed and transferred within protein complexes, monocarboxylic acids are introduced to act as energy carriers of reactive H^+^. Reactive H^+^ drives enzymatic reactions and couples the flow of energy with the flow of metabolites.

## The Steady State Versus the Energy Flow Model

**FIGURE 2:**
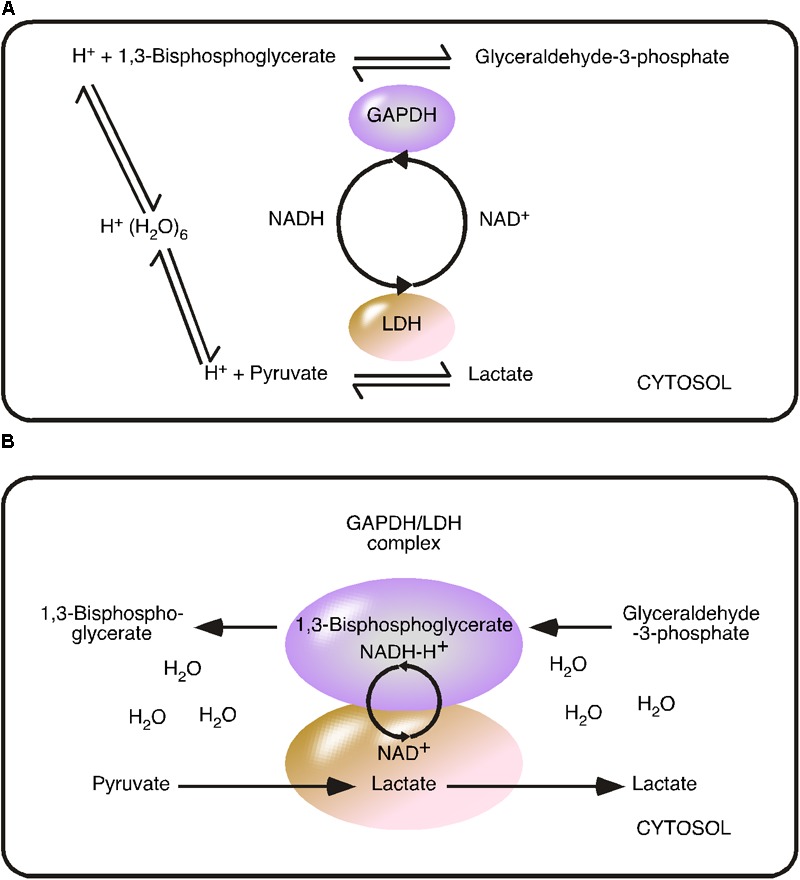
Comparison of the reversible metabolite flow and irreversible coupled flow using the glyceraldehyde 3-phosphate dehydrogenase/lactate dehydrogenase model. **(A)** Reversible metabolite flow (steady state) model: GAPDH and LDH catalyzed reversible reactions are indicated by arrows directing in both directions. The model based on cytosolic substrate concentration. Hydronium [H^+^(H_2_O)_6_] is the proton source and the coenzyme nicotinamide adenine dinucleotide (NADH/NAD^+^) acts as freely diffusing substrate. **(B)** Irreversible Coupled Flow (energy flow) model: The GAPDH/LDH complex minimizes the distance of the NADH-H^+^/NAD^+^ cycle. The concentration of the coenzyme is considered to be infinite within the complex. The coupled reaction is independent of cytosolic NADH-H^+^/NAD^+^ concentration and ratio but depends from the provision/flow. The water-free transfer of the energy carrier NADH-H^+^ turns the reaction into an irreversible reaction.

In the early 1960s, Samuel M. Rapoport and Haruhisa Yoshikawa investigated the EMP pathway in erythrocytes and understood the pathway as a flow of metabolites (Hinterberger et al., 1961; Nakao et al., 1961; Jacobasch et al., 1964). Metabolite concentrations were determined in erythrocytes (Minakami et al., 1965) and mathematical models were developed to calculate a steady state situation (Minakami and Yoshikawa, 1966; Heinrich and Rapoport, 1973; Rapoport et al., 1974). The steady state model based on cytosolic concentrations, relies on the random distribution and random movement of metabolites, or put simply ‘chaos.’ The steady state situation describes the metabolic equilibrium resulting from a continuous flow of metabolites and comprised just the eleven cytosolic enzymes of the EMP pathway, as at that time, GLUTs and MCTs were not characterized and thus could not be considered (Dubinsky and Racker, 1978; Mueckler et al., 1985) (**Figure 2**).

In this review, enzymes catalyzing glucose uptake, such as GLUTs, are considered as the first enzymes of glycolysis. This view is supported for example, by short interfering RNA knockdown experiments of GLUT family members, where knockdown drastically reduces glucose influx (Takanaga et al., 2008). Thus, driven by the concentration gradient, glucose flows into the cell and is captured by phosphorylation (Takanaga et al., 2008). It is rational that the concentration of glucose is highest at the point of entry. Thus, a theoretical model based on the random distribution and movement of molecules is inadequate to describe a flow. Although this review does not focus on regulation of glucose uptake, it is interesting to note, that Robert K. Crane characterized Na^+^-glucose co-transporters in 1961 and presented the first flux coupling in a biological system (Crane et al., 1961; Wright and Turk, 2004; Boyd, 2008).

In the steady state model, HK and phosphofructokinase were found to control glycolytic flux (Rapoport et al., 1974). At that time, glucose was considered to freely diffuse into the cell and the intracellular glucose concentration was set to the average glucose concentration (5 mM) in blood (Minakami et al., 1965; Minakami and Yoshikawa, 1966). Thus, the steady state model was based on the cytosolic substrate concentration and was not dependent on substrate provision.

In steady state situation, the state variables, such as the cytosolic pyruvate/lactate ratio, are constant. The steady state model explains known accumulation of intermediate products of glycolysis by known reversible reactions of the enzymes (Rapoport et al., 1974). We will explain the cytosolic pyruvate/lactate ratio as a result of irreversible production of lactate formed by the GAPDH/LDH complex and the reverse reaction, the oxidation of lactate to pyruvate, catalyzed by LDHs not irreversibly driven to form lactate. It has to be mentioned, that all enzymes of the EMP pathway catalyze reversible reactions, because a reversible reaction depends on both the concentration of product and substrate. Irreversible reactions, such as radioactive decay, are independent of the concentration of the product. In the steady state model, the reactions catalyzed by HK, phosphofructokinase and pyruvate kinase are virtually irreversible because the reaction equation is heavily weighted in favor of product formation and the cytosolic product concentration is not considered to reach a level sufficient to drive the reverse reaction (Stryer et al., 2002). In the early 1970s, LDH was still considered an enzyme of the EMP pathway, even under the aerobic conditions of erythrocytes. The steady state model considered the LDH-catalyzed reaction as reversible, to explain the cellular accumulation of pyruvate. Of note, GAPDH and LDH have been illustrated to be functionally connected via a NAD^+^/NADH cycle (Rapoport et al., 1974).

Recent investigations have not only demonstrated that glycolytic enzymes are organized in complexes, but also shown a direct functional connection between GAPDH and LDH (Campanella et al., 2005; Chu and Low, 2006; Svedruzic and Spivey, 2006; Kohnhorst et al., 2017). The experimentally identified proximity and functional connection between LDH and GAPDH within a complex, challenges the steady state concept at another critical point (Svedruzic and Spivey, 2006). The proximity between both dehydrogenases minimizes the distance of the NAD^+^/NADH cycle, indicating that the coenzyme, NADH, is directly consumed by LDH and immediately recovered to NAD^+^. This direct functional connection between GAPDH and LDH allows a directed transfer/flow of metabolites. The direct transfer challenges the actual concept based on random movement and random distribution, because this mechanism replaces cytosolic NADH concentration by NADH provision. As we understand it, NADH is a co-enzyme captured in the GAPDH/LDH complex.

As outlined before, a flow obeys different principles than a closed system. The flow provides the coenzyme in direct proximity of both dehydrogenases. Thus, we claim that the ‘concentration’ of the coenzyme has to be considered as infinite and independent of the cytosolic co-enzyme concentration. An infinite concentration turns the reversible reaction of LDH into an irreversible process.

For example, the reaction of a reversibly acting LDH is calculated by:

$$\left[pyruvate]\cdot [NADH]\cdot [H^{+}]⇄[lactate]\cdot [NAD^{+}\right]$$

When the concentration of NADH [mol/L] is assumed to be infinite, the reaction would proceed irreversibly. For the reverse reaction to occur the concentration of [NAD^+^] would need to be [infinity +1]. The mathematical formula (above) was created for reaction in a chaotic system. Although the above formula has been experimentally verified many times (Schwert, 1969), the formula actually supports our argument that the formation of enzyme complexes changes reversible reactions into irreversible. Thus, why should a flow obey another principle if this mathematical formula already fits?

In cells, enzymes are organized; the localization and movement of the intermediate product, here NADH, is defined by the catalyzing enzyme. Thus, the premise of the above formula (random distribution and random movement) is not adhered to. In addition, infinite concentration [mol/L] means that water is excluded (zero). By claiming that the reaction is independent of the concentration [mol/L] but depends on the provision (s^-1^), the correct term to define NADH in the mathematical formula is mol/s. A flow of metabolites is defined by mol/s.

Transferring the mathematical considerations to enzymes models, the position and movement of an intermediate product can be more precisely defined. The substrate is located within the active site of the enzyme and the substrate temporarily binds to the enzyme. At the moment of binding, the substrate is extracted from water and “solvated” by the enzyme, as shown in mathematical models of enzyme crystal structures (Ferrer et al., 2008). Thus the “concentration” of the substrate at the catalyzing enzyme is actually infinite.

However, enzymes cannot change a thermodynamic equilibrium into an irreversible reaction. A driving force or a flow of energy has to be involved to change a reaction from reversible into irreversible. Thus, we argue that the energy of the first enzymatic reaction is transferred to the co-operating enzymatic reaction and the transferred energy carrier, is reactive H^+^. In case of the GAPDH/LDH complex, we postulate that the new H^+^ is *di*solvated by NADH as NADH-H^+^.

Therefore, the suggested simplified mathematical formula to describe the LDH-catalyzed reaction in the GAPDH/LDH complex is:

$$NADH-H^{+}(mol/s)\cdot [pyruvate]\to [lactate]$$

Assuming that a single GAPDH/LDH complex captures one NADH-H^+^, then mol represents the amount of enzyme complex in the system. The transferred energy of a *di*solvated H^+^ depends on the carrier molecule. The impact of the carrier molecule on H^+^ energy transfer is best illustrated by the known acidic dissociation constants. An alternative or more precise description of energy flow should use Joule/s not mol/s. The hydration free energy of H^+^ is ∼-260 kcal/mol (∼-1090 kJ/mol) (Tawa et al., 1998; Zhan and Dixon, 2001; Hornerkamp, 2002), much greater than the hydrolysis of ATP to ADP which provides ∼-40 kJ/mol (Turina et al., 2003). The major difference between the two models is that our model of energy flow is based on postulated proton transport chains that save the energy of reactive H^+^ and irreversibly force the coupled reaction, whereas the steady state model based on a hydronium concentration [10^-7^ mol/L, pH 7].

A reversible reaction catalyzes an energy equilibrium. This catalyzed equilibrium is not a flow of energy and will therefore exclude the formation of ordered complexes. In line with this, the 4th law of thermodynamics excludes the possibility that GAPDH/LDH complexes will catalyze in both directions. Moreover, the introduced irreversible reaction of the GAPDH/LDH complex entails that LDH complexes not evolutionarily formed to catalyze the reduction to lactate, catalyze in the opposite direction, the oxidation of lactate to pyruvic acid. Thus, we consider cellular pyruvate as a product of different unidirectional driven enzymatic reactions. By doing so, the pyruvate pool can be considered as internal standard of glucose metabolism.

NADH was found to destabilize the GAPDH/LDH complex suggesting that accumulation of NADH-H^+^ opens the complex and interrupts the irreversible process (Svedruzic and Spivey, 2006). It is rational that NADH-H^+^ accumulates when the glycolytic flow is higher than the availability of the internal standard (pyruvate). Thus, the complex continuously detects/compares the glycolytic flow with the internal standard, opening of the complex interrupts GAPDH activity (glycolysis). This opening releases/pulses NADH-H^+^ as a “second messenger” to trigger proton- and redox-sensitive signaling pathways. The GAPDH/LDH complex should be considered as “receptor” permanently detecting the flow of metabolites. The steady state model discusses that HK catalyzes the most irreversible reaction and that glycolysis is controlled at the most irreversible reaction (Westerhoff et al., 2009). We introduced GAPDH/LDH catalyzed reaction as irreversible reaction in glucose metabolism. Glycolysis is not controlled at the irreversible reaction. On the contrary, the irreversible reaction controls glucose metabolism.

In the following section, the concept of energy flow will be discussed further in the context of the mLDH/MCT/PDH complex and the proton-linked MCT/carbonic anhydrase II (CAII) complex.

## Proton-Linked Monocarboxylate Transporters

**FIGURE 3:**
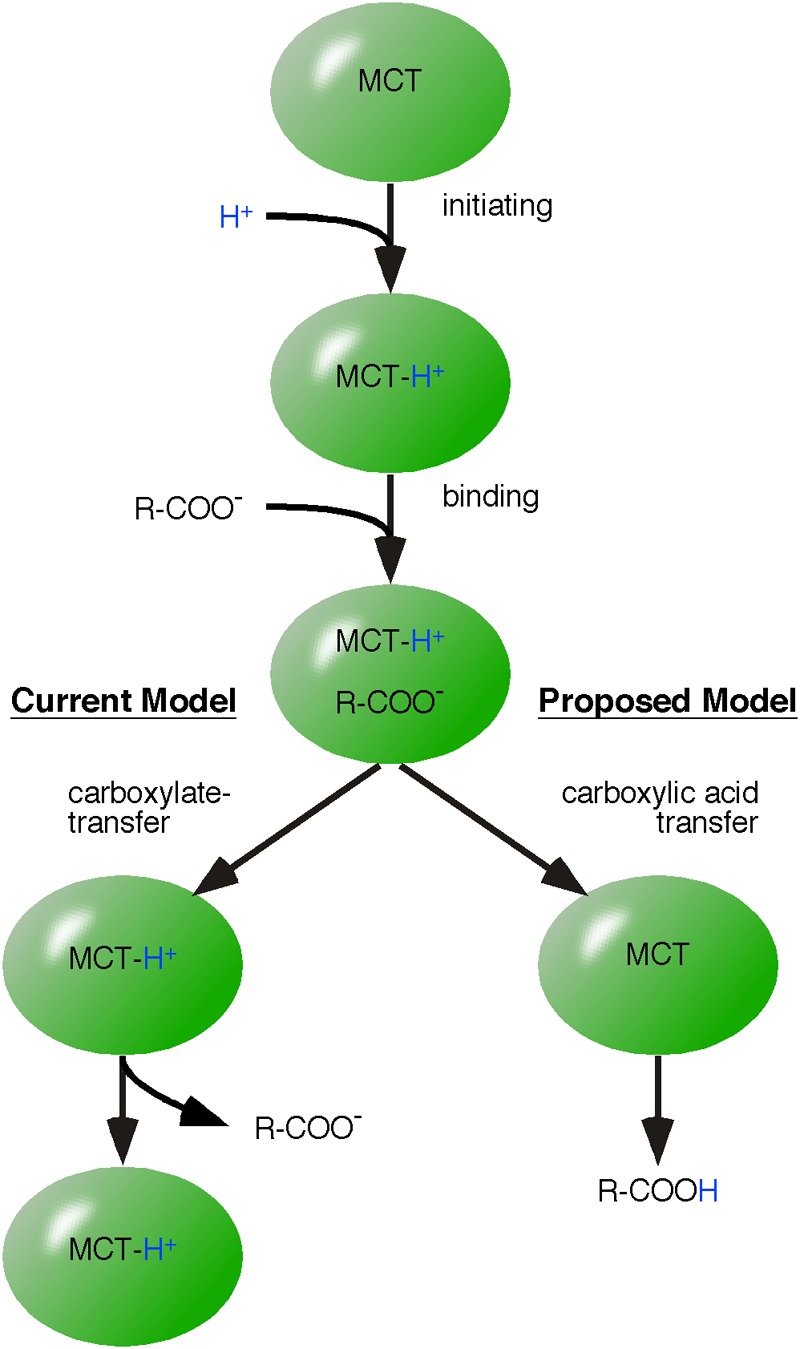
Application of the model of energy flow to monocarboxylate transporter-catalyzed membrane transfer of monocarboxylic acids. The mechanism of monocarboxylic acid transfer by proton-linked MCT is well reviewed by Halestrap (2013). Our alternative mechanism differs in the final step. The established model (left pathway) suggests that monocarboxylate is released first and MCT remains protonated. However, we postulate that H^+^ and carboxylate are released as charge-neutral carboxylic acid (proposed model, right pathway).

Monocarboxylate transporters are proton-linked monocarboxylate transporters that mediate the charge-neutral membrane transfer of monocarboxylate and H^+^ (Halestrap and Price, 1999; Halestrap, 2013). Detailed kinetic analysis of MCT1-mediated lactic acid uptake into erythrocytes revealed that transfer follows an ordered mechanism (De Bruijne et al., 1983; de Bruijne et al., 1985; Halestrap, 2013). First, a proton transfers its energy to the transporter followed by subsequent binding of the monocarboxylate anion. Next, the proton is transferred from MCT1 to the bound monocarboxylate, which is then released as lactate and H^+^ (**Figure 3**) (Halestrap, 2013).

Monocarboxylate transporters catalyze the reversible transfer of monocarboxylic acids. Reversible means that MCTs can catalyze both uptake and release of monocarboxylic acids. Reversible does not necessarily entail that one MCT complex catalyzes uptake and release of monocarboxylic acids. An essential point of this review is that a steady state situation is not the result of a reversible reaction of a single enzyme but results from enzymes embedded in complexes irreversibly catalyzing either in one or the other direction. Similar to LDH complexes, we postulate that MCTs are always organized in protein complexes and the reversible flow of metabolites is irreversibly driven by proton transport chains. Thus, we split MCT complexes into monocarboxylic acid-uptake and monocarboxylic acid-releasing complexes. This concept of splitting of MCTs into lactate and H^+^ (‘mind the proton’) uptake and export has already been shown in the oncology field (Van Hee et al., 2017). This cell-to-cell transfer of lactic acid is known as the ‘lactate-shuttle’ (Brooks, 2002; Gladden, 2004). However, the mechanisms enabling reversibly acting proton-linked MCTs to catalyze unidirectional reactions are unknown and are discussed here.

Similar to LDH complexes, the advantage of splitting MCT complexes is the vectored transport of metabolites and the possibility of independent regulation of separate uptake and release complexes. A vectored transport allows lactic acid to be continuously taken up from the environment and a directed diffusion of lactate through the cell. Directed diffusion is accomplished by MCT-releasing complexes continuously reducing the intracellular lactate concentration. This mechanism creates an intracellular concentration gradient, directed and regulated by theoretical proton-donor enzymes. Reversible-acting (single) MCTs are unlikely to perform a directed intracellular flow of metabolites, because they are unable to create a concentration gradient.

**FIGURE 4:**
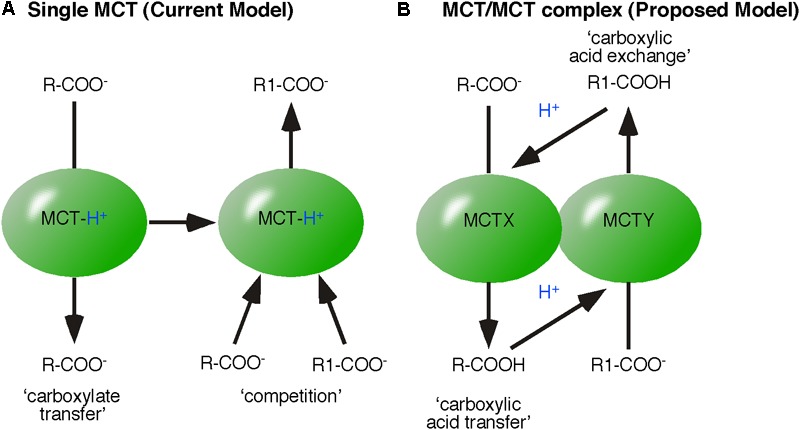
Models of monocarboxylic acid exchange. **(A)** The established model of monocarboxylic exchange suggests that MCT remains protonated and monocarboxylates compete, bind and then be transferred by MCT. **(B)** The proposed energy flow based model of monocarboxylic-exchange. MCT_X_-transfers the monocarboxylic acid (R-COOH), which then protonates MCT_Y_ (present in the same protein complex). The affinity of MCT_Y_ to R1-COO^-^ defines the exchange of R-COOH for R1-COOH.

A reversibly acting enzyme is an established template to interpret biochemical data. For example, monocarboxylate exchange was found to be faster than monocarboxylic acid transfer, leading to the interpretation that the release of monocarboxylate precedes that of H^+^. This interpretation is based on the assumption that MCT remains protonated and that by remaining protonated, the initiating (and time consuming) reaction known from monocarboxylic acid transfer is skipped. Monocarboxylates compete and bind to the protonated MCT, which then catalyzes the reverse reaction (Halestrap, 2013). Thus, a single MCT is assumed to establish an equilibrium between intracellular and extracellular monocarboxylic acid (**Figure 4A**).

The concept of energy flow based on the assumption that the binding partner of protonated MCT, is readily released as charge-neutral monocarboxylic acid. Released by MCT (acting as a proton-donor), the monocarboxylic acid dissociates in water or rapidly transfers the reactive proton to a proton/acceptor enzyme in close proximity, such as another ‘co-operating’ MCT (a proton acceptor). Thus, newly formed acids (sources of reactive H^+^) in proximity to MCTs also accelerate the initiating and rate-limiting step of monocarboxylic acid transfer by increasing the chance of proton transfer. Both models provide a theoretical mechanism explaining accelerated monocarboxylic acid exchange.

In contrast to a model of reversible acting single proton-linked MCTs, the concept of energy flow predicts the organization of MCT within protein complexes. In case of monocarboxylic acid exchange, we propose co-operation of two MCT-isoforms, X and Y (**Figure 4B**). The first MCT transfers monocarboxylic acid (R-COOH). Transferred R-COOH protonates the co-operating MCT. Protonated MCT binds R1-COO^-^ and transfers R1-COO^-^ as R1-COOH. The advantage of the energy flow model lies in the differences of affinity of the co-operating MCT subtypes. The affinity of each MCT isoform should primarily determine the nature of monocarboxylic acid exchange, because the monocarboxylate concentration is increased in proximity of the MCT by the transfer. Consequently, a reversible acting MCT should capture one monocarboxylate in a loop instead of a defined exchange.

Taken together, MCTs catalyze an equilibrium between the intracellular and extracellular concentrations of monocarboxylate/H^+^ (Poole and Halestrap, 1993). In other words, MCTs synchronize intracellular concentrations of the ligands of the ancient signaling pathway, gluconeogenesis, with the concentration in the environment, such as blood or cerebrospinal fluid. Later we will discuss that the lactate-TCA cycle is preferentially coupled with the ETC. Thus, we claim MCTs synchronize the energy situation in an organism. The concept of energy flow can only be performed by MCTs embedded in protein complexes, coupling the reversible flow of metabolites with an irreversible flow of energy. Metabolic flow is initiated and led by the provision of reactive H^+^.

Different enzymatic reactions provide reactive H^+^. Interestingly, proton-donors are CAs, which catalyze the hydration of carbonic acid anhydrate (CO_2_, carbon dioxide) and are discussed below.

## Carbonic Anhydrase/Proton-Linked Monocarboxylate Transporter Complexes

**FIGURE 5:**
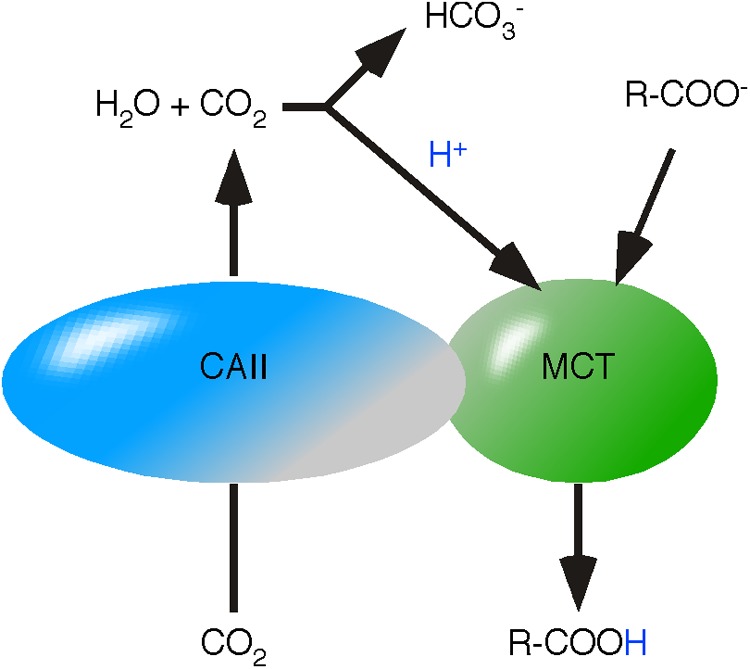
Proposed mechanism of carbonic anhydrase/monocarboxylate transporters. Carbonic Anhydrase II (CAII) and MCTs are functionally coupled in a protein complex. CAII channels CO_2_ through the membrane. Hydration of CO_2_ to H_2_CO_3_ provides an acid in close proximity to MCT. H_2_CO_3_ protonates MCT and initiates the transfer of the monocarboxylic acid.

A functional interaction between CAs and MCTs has already been demonstrated experimentally (Becker et al., 2010; Nguyen and Bonanno, 2012). CA-catalyzed hydration of CO_2_ to H_2_CO_3_ provides an acid as source of reactive H^+^ in proximity of MCT (**Figure 5**). The concept of energy flow entails that protonation of MCTs is not just the initiating step of monocarboxylic acid transfer but also defines the orientation of the monocarboxylic acid transfer. Thus, intracellular protonation of MCT initiates monocarboxylic acid release and extracellular protonation initiates the uptake into the cell. This assumption opens an interesting point in the functional connection between CA and MCT: the directed provision of H_2_CO_3_.

For a long time, CO_2_ was considered to freely diffuse through lipid bilayers, reviewed in Endeward et al. (2014). Recent data indicates that lipid bilayers retain CO_2_ and CAs are considered to function as CO_2_ channels (Itel et al., 2012). The bi-functional character of CAs was supported by co-expression of MCT4 and a hydrase-inactive CAII in *Xenopus* oocytes (Becker et al., 2010). Inhibition of hydrase activity by ethoxyzolamide or expression of the catalytically inactive mutant CAII-V143Y did not impact the increased MCT4 activity (Becker et al., 2010). This phenomenon lead to the suggestion that CAII acts as a H^+^ collecting antenna (Becker et al., 2011). We assume that lipid bilayers retain CO_2_ and CAII channels CO_2_. The resulting continuous flow of CO_2_ enhances the probability of CO_2_ auto-hydration to H_2_CO_3_ and provides reactive H^+^ independent of hydrase activity in proximity of MCT. The data additionally suggests that the channel function of CAII is sufficient to create an energy flow and hydrase activity protects the CO_2_ channel from reverse transport, guaranteeing a unidirectional flow of CO_2_.

In glycolysis, allosteric modulators are defined as molecules that have an impact on the velocity at which an enzyme-catalyzed reaction reaches equilibrium, but never change the thermodynamic equilibrium. The postulated irreversible reaction kinetics of a MCT/CA complex is not an equilibration reaction. “Modulators” of irreversible reactions, which impact the proton transfer, likely provoke an on/off regulation of complex activity. The CA activity prevents the MCT/CAII complex from catalyzing the reverse reaction, suggesting that an off switch does not entail a reverse of the catalyzed direction from export to import.

In cancer cells, endogenously expressed MCT4 catalyzes the export of lactic acid (Halestrap, 2013; Van Hee et al., 2017). However, overexpression of MCT4 and CAII in *Xenopus* oocytes promotes import of lactic acid (Becker et al., 2010). We interpret the contrast between both findings, that the endogenous proton-donor of MCT4 is not CAII. That MCT4 reverses its characterization from “export” to “import” can likely be counted to the overexpression of CAII, which dictates the direction of the catalyzed transfer. MCT4 likely takes the role of the low-expressed endogenous complex partner of CAII. This interpretation is in line with the theory of biologic robustness (Kitano, 2007). MCT4 and MCT1 carrying a CA binding domain, suggesting that another CA subtype is the preferred complex partner of MCT4. Interestingly, MCT2 does co-precipitate with CA and does not possess a CA-binding domain (Noor et al., 2015). Due to the localization of MCT2 in astrocyte endfeet and its high affinity for lactate, we assume MCT2 is involved in lactic acid export. Therefore, a predicted MCT2/PGK complex would couple the flow of metabolite in astrocytes with the release of lactic acid (Parker and Hoffman, 1967; Lauritzen et al., 2012). Thus, astrocytes could feed neuronal cells with lactic acid depending on their glycolytic flow.

## A Proton Transport Chain

**FIGURE 6:**
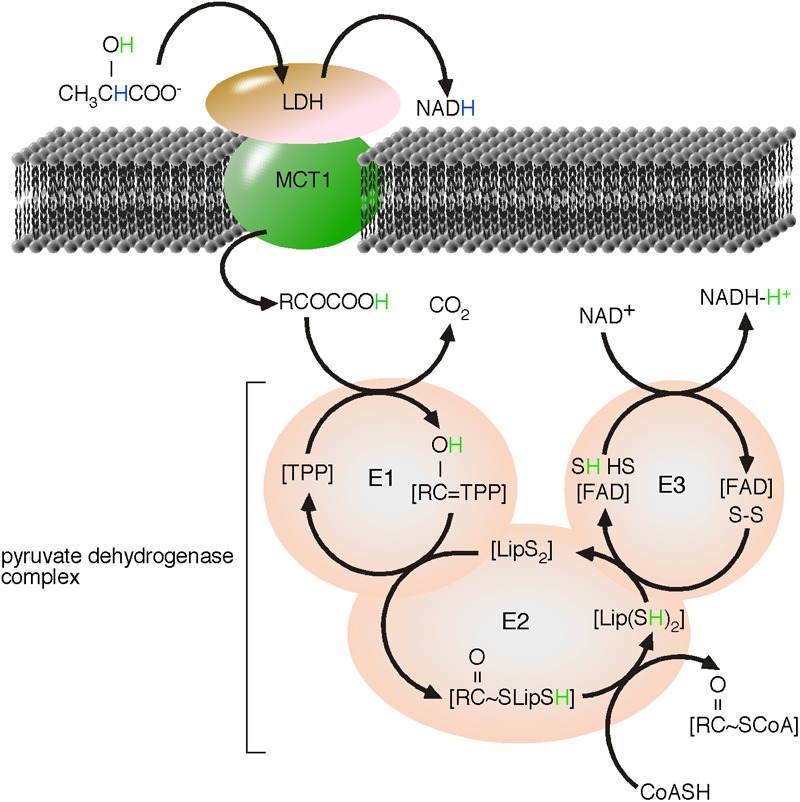
Proposed proton transport chain of the lactate-driven pyruvate dehydrogenase complex. LDH catalyzed the oxidation of the α-hydroxyl group of lactate to the α-keto-group of pyruvate. Nicotinamide adenine dinucleotide (NAD^+^) is reduced to NADH and a reactive H^+^, originally from the α-hydroxyl group of lactate, is formed. In absence of water, pyruvate *di*solvates the new reactive H^+^ as pyruvic acid. The monocarboxylate transporter, MCT1 catalyzes the charge-neutral membrane transfer of pyruvic acid. The substrate of the α-ketoacid, PDH, is pyruvic acid (Das et al., 1961). Within the PDH complex, H^+^ takes active part during all reaction and finally ends, *di*solvated by NADH. We modified the figure from L. Reed, by highlighting the H transport, adding the mLDH/MCT1-complex as first enzymes of the lactate-driven PDH complex and indicating that NADH *di*solvates H^+^ (Reed and Hackert, 1990).

Earlier in the review, reactive H^+^ was introduced as an energy carrier, driving the flow of energy. The tentative fourth law of thermodynamics describes that a flow of energy is sufficient to form ordered structures (Morowitz, 1992; Jorgensen, 1999). Considering H^+^ as energy, the concept predicts the formation of proton transport chains that can mediate a directional transport of energy (*di*solvated H^+^) within ordered structures. PDH is the first enzyme of the TCA cycle and is embedded in the PDH complex, which is localized to the mitochondrial matrix. The PDH complex is known to catalyze the coupled reactions; the decarboxylation of pyruvate and the covalent binding of hydroxy-ethyl to thiamin pyrophosphate (Tpp). The intermediate product is hydroxyl-ethyl:Tpp, the end product is CO_2_ (Stryer et al., 2002). Relatively new members of the PDH complex are MCT1 and mLDH (Brooks et al., 1999; Hashimoto et al., 2008). Both are essential enzymes of the complex because, based on our concept, they catalyze the formation and directional transport of pyruvic acid (**Figure 6**).

A critical point in the model of energy flow is likely the acceptance of the formation of monocarboxylic acids as intermediate products or the existence of carriers of reactive H^+^. Organized in a complex, it is reasonable to argue that the product of MCT1 should be the substrate of PDH. In 1961, Lester Reed wrote CH_3_COCOOH (pyruvic acid) in the PDH-catalyzed reaction equation (Das et al., 1961). In 1995, Lester Reed discussed PDH as member of the mitochondrial located α-ketoacid dehydrogenase family (Reed and Hackert, 1990). The following equation represents the CoA- and NAD^+^-linked oxidative decarboxylation of α-ketoacids, modified from Reed and Hackert (1990).

$$RCOCOOH\;+\;CoA-SH\;+\;NAD^{+}\to RCOSCoA\;+\;CO_{2}+\;NADH-H^{+}$$

Unfortunately, H^+^ is usually removed from the reaction equation giving:

$$RCOO^{-}+\;CoA-SH\;+\;NAD^{+}\;\to \;RCOSCoA\;+\;CO_{2}\;+\;NADH$$

We believe that removal of H^+^ from the reaction equation has created a fatal pitfall in biology, as the substrate of α-ketoacid dehydrogenase turns from acid to carboxylate. Not ‘minding the proton’ has consequently lead to α-ketoacid dehydrogenases being turned into α-ketocarboxylate dehydrogenases, such as PDH. Thus, substrate of PDH is pyruvic acid (Das et al., 1961). Pyruvic acid is charge neutral and can be transferred through the IMM without creating an electric potential. The PDH complex is part of a proton transport chain, where reactive H^+^ is transferred by many steps from pyruvic acid to give NADH-H^+^. The natural tendency of H^+^ to be *di*solvated means that H^+^ must to be considered to be bound to a carrier molecule, thereby excluding its removal from chemical equations. Removing H^+^ from the chemical reaction not only prevents the membrane transfer of the substrate of the α-ketoacid dehydrogenase, but also all enzymatic steps of the PDH complex based on the existence and the transfer of this H^+^ (**Figure 6**). The PDH complex represents a proton transport chain. Pyruvic acid forms a penta-ring structure to *di*solvate H^+^. The *di*solvated hydrogen is already orientated to the O atom of the α-C of pyruvic acid and is essential for the PDH-catalyzed covalent binding of this *di*solvated H^+^ to the O atom. The postulated formation of monocarboxylic acids by mLDH and MCTs and the existence of proton transport chains are thereby supported by the nature of H^+^ to be *di*solvated. The resulting coordination of H^+^ in a penta-ring-structure promotes optimized energy transfer by skipping hydration/dehydration of reactive H^+^, and finally PDH is member of the α-ketoacid dehydrogenase family, rather than a α-ketocarboxylate dehydrogenase.

## The TCA Cycles

Mitochondrial located processes were introduced as regulated crosscuts of glycolysis. The “pressure” of the flow of energy, or the concentration of lactate in the intra-mitochondrial space, is surely one factor regulating the activity of the mLDH/MCT1/PDH complex. The intra-mitochondrial lactate concentration depends on two distinct pathways: glycolysis (uptake of glucose) and the uptake of lactic acid by MCTs. Considering the mitochondrial TCA cycle as a crosscut predicts a regulated uptake of the PDH substrate, pyruvic acid. Uptake of pyruvic acid has to be coupled with feedback mechanisms indicating activity/requirement of the TCA cycle.

Primary indicators of enzyme-catalyzed reactions are end products of the process. The end product of the TCA cycle is CO_2_. If we assume that the IMM retains CO_2_, mitochondrial located CAs will channel CO_2_ and transfer reactive H^+^ to a pyruvate carrier, which will then catalyze the charge-neutral membrane transfer of pyruvic acid, the substrate of PDH (Bricker et al., 2012; Herzig et al., 2012).

The TCA cycle includes three protein complexes catalyzing the irreversible decarboxylation of metabolites: PDH, isocitrate dehydrogenase and α-ketoglutarate dehydrogenase complex (Lionetto et al., 2016). If we assume that the membrane transfer of pyruvic acid into the mitochondrial matrix is controlled by the end product (CO_2_) of these dehydrogenases and driven by flow of reactive H^+^ into the mitochondrial matrix, “The TCA cycle” has to be split into lactate-driven and pyruvate-driven TCA cycles, because mLDH already oxidizes lactate to pyruvic acid whereas cytosolic pyruvate needs a reactive H^+^ to be charge-neutral transferred as pyruvic acid.

If the IMM retains CO_2_ and dehydrogenases directly interact with a CO_2_ channel, the pyruvate-TCA cycle can be further divided into PDH-, isocitrate dehydrogenase- and α-ketoglutarate dehydrogenase-regulated pyruvate-TCA cycles. The assumption that three different dehydrogenases are able to regulate substrate supply enables the pyruvate-TCA cycle to detect the requirements of a variety of anabolic reactions connected with the TCA cycle.

mLDH/MCT1/PDH are the first enzymes of the lactate-TCA cycle, where mLDH catalyzes the formation of pyruvic acid. In contrast to the pyruvate-TCA cycles, the substrate of PDH is already provided and not a regulated step. A continuous supply of pyruvic acid depends on the recovery of mLDH/NADH to mLDH/NAD^+^. As discussed by Rogatzki et al. (2015) a variety of mitochondrial located mechanisms can be functionally connected to redress the mLDH/NADH to mLDH/NAD^+^ balance. This review follows the line of thought that the end product indicates the requirement of the process and that mitochondrial located processes are also end product-regulated crosscuts. The end product of the ETC is NAD^+^. A reasonable mechanism regulating ETC activity is coupled with recovery of mLDH activity. NAD^+^/NADH exchange would immediately couple the end product of the ETC with the supply of pyruvic acid. The suggested NAD^+^/NADH exchange still excludes regulatory feedback from the lactate-TCA cycle. The proposed NAD^+^/NADH-exchange transfers the cation (NAD^+^) through the IMM. An obligate charge-neutral membrane transfer of NAD^+^ is accomplished by a proposed NAD^+^/NADH-H^+^ exchange. Reactive H^+^ from the lactate-TCA cycle would act as driving force of NAD^+^-membrane transfer. The assumed NAD^+^/NADH-H^+^ exchange merges the regulation of the lactate-TCA cycle with ETC. Excellent candidate proteins connecting ETC- and TCA-regulation are NAD^+^ transporters, given that the transfer of NAD^+^ through the IMM is charge-neutral (Todisco et al., 2006).

## Discussion

The glycolytic pathway is for first time introduced as a flow of metabolites driven by an irreversible flow of energy. The concept includes the eleven cytosolic enzymes of the EMP pathway as well the enzymes mediating the uptake of glucose and the release of lactic acid. Considering glycolysis to be driven by an irreversible flow of energy challenges all common understanding(s) of the glycolytic pathway at their roots.

The review integrates fundamental principles into glycolysis, such as the principle of mass conservation and that membrane transfer of ions is blocked by the creation of an electric potential.

**FIGURE 7:**
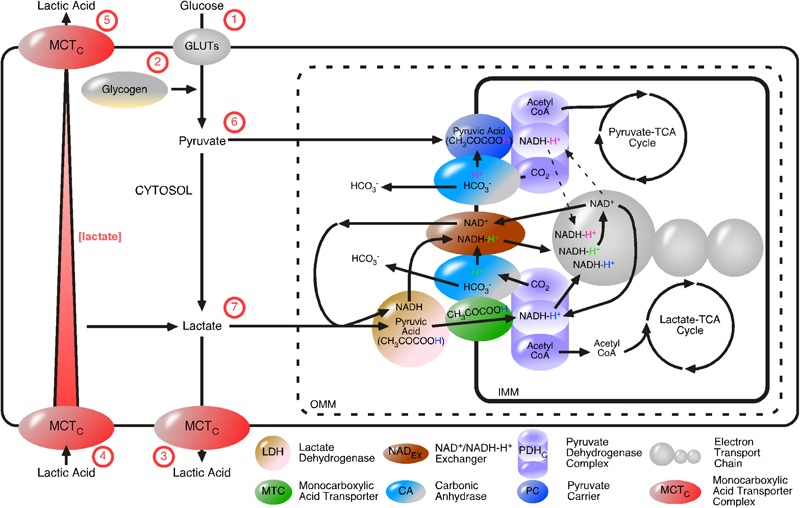
Proposed model of proton-driven monocarboxylic acid import and export. GLUTs and proton-linked monocarboxylate transporter complexes (MCT_C_s) are added to the glycolytic flow. MCT_C_s regulate lactate flow, by directed import and export of lactic acid. The irreversibly GAPDH/LDH-catalyzed formation of lactate is independent from cytosolic lactate concentration. Cell membrane MCT_C_s are composed of a MCT and a proton-donor protein, for examples, carbonic anhydrase (CA) or phosphoglycerate kinase. Intracellular lactate concentration is dependent on glycolysis and the transport pathways. The irreversible nature of the pathways makes their flow independent from intracellular lactate concentration, but dependent on energy provision (H_C_). Glycolysis depends on (1) the uptake of glucose via GLUTs or (2) glycogen breakdown and is completed by the (3) release of lactic acid by MCT_C_s. Lactic acids are (4) taken up or (5) released by cells is directed by the postulated proton-donor proteins of the MCT_C_s. Pyruvate and lactate are considered as intermediate products of glycolysis. (6) Pyruvate diffuses from the cytosol through pores located in the OMM and is transported as pyruvic acid across the IMM via the pyruvate carrier. Pyruvic acid and NAD_C_ are then converted to acetyl CoA, NADH-H^+^ and carbon dioxide (CO_2_) by the action of the pyruvate dehydrogenase complex (PDH_C_). Acetyl CoA enters the pyruvate-driven TCA cycle. It remains determined if NADH-H^+^ feeds into the ETC (dotted line with arrows) and CA uses CO_2_ to generate a reactive H_C_ (that is used to complete the charge-neutral transfer of pyruvic acid across the IMM) and HCO_3_ or if the pyruvate-TCA-cycle just feeds anabolic TCA-coupled processes. Lactate generated by glycolysis or taken up by the cell, diffuses through pores located in the OMM, converted to pyruvic acid by LDH and then be transported across the IMM by MCT (7). The PDH_C_ converts pyruvic acid and NAD_C_ to acetyl CoA (to feed the lactate-driven TCA cycle), CO_2_ (to feed CA) and NADH-H^+^ (to feed the ETC). As before, CA catalyzes the reaction between CO_2_ and H_2_O to give rise to HCO_3_^-^ and H^+^. The reactive H^+^ is then *di*solvated by LDH-generated NADH, the subsequent NADH-H^+^ is passed from the NAD^+^/NADH-H^+^ exchanger (NAD_EX_) to power the ETC. An excellent candidate MCT_C_ coupling the rate of glycolysis with the cellular export of lactic acid is the combination MCT2/phosphoglycerate kinase. Excellent candidate MCT_C_s coupling ETC-rate with lactic acid import or export are MCT1/CAII and MCT4/CA, respectively.

Glycolysis starts with the regulated uptake of glucose and ends with the regulated release of lactic acid (summarized in **Figure 7**). The introduced irreversible character of the pathway, especially the GAPDH/LDH complex, entails that other “glycolytic” LDH complexes, such as the mLDH/MCT1 complex, catalyze in the opposite direction at the same time, in the same cell. Our model of unidirectional acting enzyme complexes provides regulative advantages that the steady state model does not provide. The steady state model was developed on the basis of cytosolic concentration and enzymes catalyzing an equilibrium. Enzymes catalyzing an equilibrium cannot move substances through cells. The advantage of considering MCTs to always be co-localized with a proton-donor enzyme, is that it allows the movement of molecules through cells, because H^+^ provides the power to create a concentration gradient of lactate by defined import and export of lactic acid (**Figure 7**). We suggested that there are two distinct regulative mechanisms for lactic acid export. MCT2/PGK couples lactic acid export with the glycolysis rate. MCT1/CAII couples lactic acid export with the ETC. In **Figure 7** MCT complexes are illustrated without enzyme-subtype specific information.

In the 1960s, Harold J. Morowitz postulated, that the flow of energy through a system is sufficient to form ordered structures (i.e., organize the system) (Morowitz, 1992). Transferring the tentative fourth law of thermodynamic to the molecular level of a cell suggests that protein complexes of the glycolytic pathway are formed by energy flow.

We discussed that ordered structures involved in and evolutionarily formed by a flow of energy, obey principles different to isolated enzymes. The proximity of organized enzymes allows a direct substrate transfer with the consequence that the kinetic of the enzyme is independent of cytosolic substrate concentration. Based on enzyme crystal structures, substrate concentration is infinite. The substrate is bound or solvated by the enzymes. The assumed absence of water abolishes hydration of new H^+^ and allows energy transfer. This concept leads to the postulation of proton transport chains.

The formation of ordered structures is generally accepted as fourth law of thermodynamics, but the reason and consequence behind the formation are a matter of debate and likely be dependent on the system discussed. Furthermore, maximum power or maximum flux is considered to be the consequence of ordered structures (Odum, 1995; Jorgensen, 1999). The system, or cell, has developed under the evolutionary pressure of provision of food (glucose). Protein complexes minimize the distance of energy transfer at a molecular level, which changes reversible processes into irreversible and allow detecting of metabolite flow.

We suggest that protein structures are developed to optimize the metabolism of glucose in a way that energy and metabolite requirement is covered and the loss of energy (anergy/heat) is minimized, in short, maximum power with minimum flux. For instance, as the fermentation processes emit heat (Brown, 1901), it is tempting to suggest that the development of ordered structures participated into the development of endotherms. Body temperature is a strictly controlled parameter, indicating that heat-development by hydration of H^+^ has to be decoupled from glycolytic flow.

The established LDH-truncated model of the EMP pathway entails a different assumption to cover the removal of LDH activity and the presence of lactate in cells, blood and CSF. LDH-truncated model does not include the GAPDH/LDH complex, which immediately recovers GAPDH-NADH-H^+^ to GAPDH-NAD^+^. Instead of LDH-catalyzed redress of NADH, continuously formed cytosolic NADH is considered to fuel oxidative phosphorylation (ETC) to maximize ATP production (Vander Heiden et al., 2009). The created H^+^ is not considered.

We claim that transport of molecules is promoted by affinities and driven by energy flow. This concept is not in line with a mechanism suggesting diffusion of NADH to mitochondrial processes. This mechanism includes the diffusion of NADH from the cytosol into the mitochondrial matrix and recycling of NADH as NAD^+^ into the cytosol. This cycle, not only twice encounters two lipid bilayers, but diffusing NADH is in competition with any cytosolic located NADH-dependent processes.

The LDH-truncated model differentiates between “aerobic” and “anaerobic” glycolysis to explain the existence of lactate. Yet, Pasteur reported that yeast produce more fermentation products under anaerobic conditions than aerated cells, Pasteur Effect (Pasteur, 1861). The Pasteur Effect was determined for facultative anaerobic single cells and transferred to obligate aerobic cells embedded in an organism to explain the enhanced release of lactate (and H^+^) in tumor or active muscle cells by “anaerobic” glycolysis.

In unicellular organisms, the end products of glucose metabolism can simply diffuse away, but once multicellular organisms had evolved, they had to devise methods for safely dealing with them (Buck and Levin, 2011). Metabolites, especially the end products of glucose metabolism, lactic acid, and carbon dioxide, are considered as primary messengers indicating and regulating cellular homeostasis. Once released in the environment, they indicate the metabolic activity of a single cell to the whole organism. The presented model introduces glycolysis as a unidirectional, continuously flowing pathway. Intermediate products of the pathway, such as pyruvate and lactate, are substrates of metabolic crosscuts. Pyruvate and lactate are substrates of distinct TCA cycles and based on the presented theoretical regulative mechanism, the lactate-TCA cycle is preferentially coupled with the ETC.

Seventy five years ago, Otto Warburg recognized that malignant cancer cells typically displayed high rates of glycolysis even when fully oxygenated (Warburg, 1930; Gatenby and Gillies, 2004). “Aerobic” glycolysis in tumor cells is associated with increased expression of HK and up-regulation of GLUTs across the plasma membrane (Bustamante et al., 1981; Geschwind et al., 2004). Tumor mitochondria are fully functional with regard to respiration and ATP synthesis (Nakashima et al., 1984). This data fits perfectly into the model of energy flow. An increased substrate-uptake rate (up-regulation of GLUTs across the plasma membrane) results in an increased flow of energy and increased production and release of lactic acid. The resultant increased release of lactic acid, is essential to indicate the organism that, for example tumor cells, have increased lactate-TCA/ETC activity. This can only be explained by considering TCA cycle as crosscuts of the glycolytic pathway and an alternative pathway for metabolites.

Increased lactate in the blood enhances lactic acid up-take and, based on our model, synchronize the energy situation in an organism (Poole and Halestrap, 1993). This phenomenon is observed during sport activity. Based on our model, a working muscle will produce a temporary increase of lactate-TCA cycle and ETC activity. During strenuous exercise, consumption of lactate (and oxygen) is increased and lactic acid is increased released from muscle cells to indicate enhanced energy turnover to the organism. It is tempting to suggest, that the end product of glycolysis, lactic acid, is released as indicator of the glycolytic flow. One glucose molecule is metabolized to two lactic acid molecules. In order to indicate the metabolism of one glucose molecule, it is reasonable to suggest that one of the two lactic acids is released as an indicator to synchronize the energy situation of an organism. Of course, muscle cell re-uptake of lactic acid is increased by accelerated CO_2_ production. Re-uptake of lactate is discussed to be coupled with the release of CO_2_. Release and re-uptake of lactic acid is essential to synchronize the energy situation in an organism. Thus, an increased lactate level during strenuous exercise is considered to be synchronization of glycolysis rates in the organism. An increased lactate level during hypoxia is considered to be the effect of decreased re-uptake of lactic acid. Moreover, lactate can be detected by G protein-coupled receptors and is known to control breathing, delay neurodegeneration and lactate detection is crucial for cancer survival (Roland et al., 2014; Chang et al., 2015; Morland et al., 2017).

Until now, this review has provided mechanisms whereby glycolysis can detect and transmit the energy flow of a cell to the organism. We have failed to discuss the glycolytic pathway as an ancient and ubiquitous pathway of regulating life. This function of the glycolytic pathway has already been published following an investigation of sperm capacitation and the characterization of soluble adenylyl cyclase (sAC) (Hess et al., 2005; Xie et al., 2006). Applying our model on sperm capacitation, sperm adapt their flow of energy to the extracellular indicators of energy flow. High carbonic acid concentration indicates high mitochondrial (lactate-TCA/ETC) function. Sperm metabolism adapts to high energy flow by breakdown of glucose from intracellular stores. The increased energy provision, triggered by carbonic acid/CO_2_, is coupled to a signaling cascades leading to capacitation. sAC was classified as an ancient pH-sensitive bicarbonate sensor, that is embedded in protein complexes located in the cytosol, mitochondria and nucleus (Wuttke et al., 2001; Zippin et al., 2003). sAC is also considered as a metabolic AC, sensing and regulating the flow of metabolites/energy. *Ex vivo* characterization of isolated sAC clearly demonstrated that sAC activity increases with the concentration of bicarbonate and decreases with the concentration of H^+^(H_2_O)_6_ in the buffer, leading to the classification of sAC as pH-sensitive bicarbonate sensor (Chen et al., 2000; Levin and Buck, 2015). This interpretation of sAC function is based on the steady state concept and cytosolic concentration, however, a regulation of a flow can barely be performed to detect fluctuations within the relative constant cellular bicarbonate-buffer. Additionally, this experimental design assumes that other regulative parameters remain constant. CO_2(g)_ carries two polarized bonds. The polarized bonds, or the partial charge of the carbon and the two oxygen atoms means that CO_2_ does not freely diffuse through lipid bilayers and additionally the solubility of CO_2(g)_ in water should increase with ionic strength. Like dissolves like (Wypych, 2001). This assumption opens the possibility that sAC can detect CO_2(g)_. Thus, it is tempting to suggest that CO_2(g)_ competes with sAC-bound bicarbonate (Saalau-Bethell et al., 2014). Embedded in protein complexes, it also tempting to suggest that sAC detects the release of reactive H^+^ converting sAC-bound bicarbonate to carbonic acid (Saalau-Bethell et al., 2014).

The power of the presented model lies in splitting metabolic reactions into irreversible-acting protein complexes. These complexes work by permanently detecting the flow of glucose and lactic acid and transmit changes in the flow as NADH and H^+^ pulses. We argue that single cells adapt their homeostasis to the metabolites in the environment via ancient signaling pathways, whereas multicellular organisms synchronize their energy situation via ancient signaling pathways. The evolutionary development of differently regulated GLUTs and MCTs and their specific subcellular localization is likely an essential factor in the development of multicellular organism. In organisms, cells communicate by hormones, neurotransmitters or cytokines. Any detectable transmitter indicates a change of the environment and entails a change of the energy state of the cell. Specific signal pathways guide the cell to adapt to the environmental change. The “most simplified” understanding of cell-cell communication in multicellular organism is likely to consider the ancient signaling pathways of glucose metabolism as “operating system” and hormones, neurotransmitter, cytokines and so on as evolutionary developed “input routines.” Many chronic diseases, including diabetes, hypertension, schizophrenia, multiple sclerosis are coupled with inflammatory processes and dysregulated glucose/lactate/H^+^ levels in the environment or transmitter levels (Juraschek et al., 2013, 2015; Rowland et al., 2016; Pucino et al., 2017). An imbalance between glucose and insulin, or just high glucose increases release of proinflammatory cytokines (Clay et al., 2011). It is tempting to suggest that cells just communicate about their metabolism and that chronically dysregulated metabolite levels in the environment permanently stresses specific protein complexes. This stress likely impacts protein kinase A-, protein kinase C- and redox-sensitive pathways controlling basal gene transcription and basal enzyme activity. Considering glycolysis and gluconeogenesis as a regulatory pathways, the huge variety of mRNA, proteins, peptides and neurotransmitter already identified to be dysregulated by chronic diseases can be sorted into dependently or independently controlled processes by the metabolic profile of the environment or the pulses of H^+^ and NADH (Clay et al., 2011; Davalieva et al., 2016; Kahl et al., 2016; Schubert et al., 2016).

Additionally, enzymes such as glycogen synthase kinase-3 or COMT already connected with the glucose metabolism are now connected with ancient signaling pathways detecting metabolite flow (Veser and Martin, 1986; Emamian, 2012). Metabolite flow is detected by different complexes located at membranes or the cytosol. COMT is also located at membranes and the cytosol, but just the genetic knock-down of membrane bound COMT creates a schizophrenic-like phenotype in mice (Tammimaki et al., 2016). They are many pathways to psychosis (Seeman et al., 2005). Future work will demonstrate how many of them are ancient.

## Author Contributions

DR and GC formulation of theory, drafting the article, and revising it critically. WM drafting the article and revising it critically.

## Conflict of Interest Statement

The authors declare that the research was conducted in the absence of any commercial or financial relationships that could be construed as a potential conflict of interest.

## Abbreviations

CAC: carbonic anhydrase
COMTC: catechol O-methyltransferase
EMPC: Embden–Meyerhof–Parnas
ETCC: electron transport chain
GAPDHC: glyceraldehyde 3-phosphate dehydrogenase
GLUTsC: glucose transporters
HKC: hexokinase
IMMC: inner mitochondrial membrane
LDHC: lactate dehydrogenase
mLDHC: mitochondrial LDH
MCTC: monocarboxylate transporters
NAD^+^C: oxidized nicotinamide adenine dinucleotide
NADHC: reduced nicotinamide adenine dinucleotide
OMMC: outer mitochondrial membrane
PDHC: pyruvate dehydrogenase
PGKC: phosphoglycerate kinase
TCAC: tricarboxylic acid
